# Sobre o reducionismo na pesquisa do trauma

**DOI:** 10.1590/0102-311XPT226524

**Published:** 2025-01-27

**Authors:** Ramon Reis dos Santos Ferreira, Francisco Ortega

**Affiliations:** 1 Universitat Rovira i Virgilli, Tarragona, España.; 2 McGill University, Montreal, Canada.; 3 Instituciò Catalana de Recerca i Estudis Avancats, Barcelona, España.

Nos últimos anos, o interesse pelo mapeamento epidemiológico das condições secundárias a acontecimentos potencialmente traumáticos explodiu ^1^, levando a um conhecimento abrangente sobre a distribuição, o impacto e os fatores de risco e proteção a que estão submetidas diversas populações. Conceitos como os de prevalência à exposição, prevalência total ou risco condicional se sobrepuseram para moldar diferentes metodologias de apuração das repercussões culturais, individuais e societárias do trauma e, se antes o debate sobre a traumatização permanecia confinado à classificação da malignidade dos eventos ou da adaptabilidade comportamental das respostas, hoje o repertório do campo se complexificou para incluir as determinações culturais ^2^, as trajetórias de recuperação ^3^ e as mudanças epigenéticas em sujeitos traumatizados ^4^. Indexada à inespecificidade da sintomatologia e ao regime de exceção concedido às categorias psicopatológicas que gozam de um evento etiológico circunscrito como critério diagnóstico *sine qua non* ^5^, a racionalidade traumática se propaga, então, para o território dos transtornos depressivos, ansiosos e, mesmo, para o espectro das psicoses.

Tal movimento, embora efetivamente remonte à emergência do campo dos estudos sobre o estresse traumático, ainda tem seus antecedentes históricos e epistemológicos pouco reconhecidos ou mal delineados. Alguns dos seus mais importantes tratados, como os excelentes trabalhos de Young ^6^ e Fassin & Rechtman ^7^, respectivamente interessados na descrição dos contextos políticos, sociais e institucionais da elaboração do transtorno de estresse pós-traumático (TEPT), ainda que destaquem as dimensões fundamentais da memória, da confissão e do legado de suspeição de vítimas de trauma, não explicam as determinações epistêmicas pelas quais passa a memória traumática do século XIX, que uma vez emancipada da traumatização física do choque cirúrgico, vem a retomar uma conformação essencialmente somática ao se dissipar no vocabulário bioquímico do estresse. Tal análise, realizada neste e em outros ensaios publicados por esses autores ^8^ ^,^ ^9^ ^,^ ^10^, procura preencher essa lacuna e, portanto, indagar por quais meios os conceitos de inspiração mentalista outrora utilizados na interpretação dos fenômenos traumáticos passaram a ser incorporados por abordagens de matriz neurobiológica que, ao reduzir o trauma às suas categorias de interesse (e.g., correlatos neurais, regiões anatômicas, concentrações metabólicas), eliminam não só a pertinência de doutrinas cuja racionalidade caem fora de seu instrumental discursivo, mas, sobretudo, interditam a análise dos aspectos subjetivos e fenomênicos do sofrimento que interessam a estas.

Nesse ponto, talvez seja importante uma breve análise do reducionismo, a partir de um fenômeno mais elementar que o trauma.

Já foi dito que a análise dos mecanismos anatômicos e funcionais responsáveis pela experiência consciente constituiria o maior desafio da neurociência moderna ^11^. A envergadura dessa tarefa, que efetivamente corresponde à resolução da herança legada ao pensamento ocidental pelo dualismo cartesiano, representa a eliminação do intervalo entre os eventos que transcorrem nos planos físico e fenomênico da experiência. Porém, reduzir a consciência humana a seus correlatos cerebrais não constitui o mesmo que reduzir a água ao H_2_O, o relâmpago à descarga elétrica, o carvalho ao hidrocarboneto ou o gene ao DNA. Há algo mais na redução psicofísica do que se procede nas reduções físico-químicas alcançadas em outros problemas apresentados pelos reducionistas ^12^ ^,^ ^13^. Ainda que os mecanismos causais para uma descrição física da percepção visual possam caber na ideia de que a visão é o resultado da discriminação e categorização de ondas eletromagnéticas por um dado sistema visual, nada explicaria por que esse processamento é acompanhado pela experiência de sentir o vermelho vivo ou o azul profundo ^14^. Não dispomos de nenhum modelo físico confiável para o funcionamento mental, porque nenhum modelo de redução, quer metodológico, semântico ou ontológico ^15^, captura o caráter subjetivo da experiência. Isso significa que tanto a direção, quanto a ordem de grandeza desses métodos permitem que a redução seja compatível com a ausência do fenômeno que ela intenciona reduzir, pois este não participa da estrutura da própria inspeção materialista ^16^. Desse modo, todos os métodos que assim procedem avaliam o mental independentemente da dimensão subjetiva da experiência, uma vez que esta é a principal unidade expropriada pela operação redutiva.

Portanto, para o debate do reducionismo, a natureza ou a coerência das explicações de um dado fenômeno parece menos relevante que o poder de um determinado campo/paradigma em chancelar suas próprias práticas discursivas e métodos de produção de verdade. Lembremos que as condições de validade para a utilização de evidências na formulação das hipóteses em psicanálise já vêm sendo exaustivamente avaliadas nos últimos 50 anos em torno de noções como o “argumento de registro” que, em geral, discute se a tentativa psicanalítica de associar o sucesso terapêutico a formulações teóricas se adequa suficientemente às prescrições do indutivismo eliminativo ^17^. Logo, independentemente se a traumatogênese for o resultado das experiências descritas nas teorias freudianas da sedução ou da fantasia, ou de uma rede córtico-límbica envolvida no funcionamento recíproco da amígdala, do córtex pré-frontal medial e do hipocampo ^18^, a questão fundamental parece ser a de avaliar se de tal multiplicidade teórica redundam múltiplos acervos práticos, apoios institucionais e paradigmas metodológicos.

A ambição de submeter leis e fatos empíricos de uma determinada disciplina às leis e aos fatos empíricos de outra parece ser tão antiga quanto a aposta da filosofia mecânica do século XVII em reduzir todos os eventos físicos às interações de contato local entre partículas materiais impenetráveis ^15^. Tal programa descreve uma metodologia calcada na operação explicativa interteórica mediante a qual determinada teoria reduzida encontra seu “esclarecimento” definitivo em uma teoria redutora pressuposta como mais fundamental. Assim, desde uma perspectiva filosófica, a redução representa uma relação entre hipóteses cujo caráter explicativo resulta da presunção de superioridade da teoria redutora sobre a reduzida ^19^ e do pretenso isomorfismo entre a entidade e os predicados dos termos reduzidos, quer pela maior sistematização, quer pela amplitude explicativa superior. No campo do estresse pós-traumático, muito além de bradar pela pluralidade de modelos de pesquisa, devemos nos perguntar se a admissão de determinados paradigmas incorre (ou não) na eliminação progressiva de outras formas de representação da natureza e do sofrimento. O fato das teorias neurocientíficas contemporâneas do psicotraumatismo resultarem das hipóteses psicodinâmicas de Pierre Janet no século XIX nos adverte a esse respeito.

O problema do trauma - e o de qualquer experiência - será, sempre, uma questão de quantidades e qualidades.
